# Bacteriophages as potential new mammalian pathogens

**DOI:** 10.1038/s41598-017-07278-6

**Published:** 2017-08-01

**Authors:** George V. Tetz, Kelly V. Ruggles, Hua Zhou, Adriana Heguy, Aristotelis Tsirigos, Victor Tetz

**Affiliations:** 1 Human Microbiology Institute, New York, NY 10027 USA; 2 Department of Medicine, New York University School of Medicine, New York, NY 10016 USA; 3 Applied Bioinformatics Laboratories, New York University Medical Center, New York, NY 10016 USA; 4 Department of Pathology, New York University School of Medicine, New York, NY 10016 USA; 5 Laura and Isaac Perlmutter Cancer Center, New York University School of Medicine, New York, NY 10016 USA; 6 Genome Technology Center, Division of Advanced Research Technologies, NYU School of Medicine, New York, NY 10016 USA

## Abstract

Increased intestinal permeability and translocation of gut bacteria trigger various polyaetiological diseases associated with chronic inflammation and underlie a variety of poorly treatable pathologies. Previous studies have established a primary role of the microbiota composition and intestinal permeability in such pathologies. Using a rat model, we examined the effects of exposure to a bacteriophage cocktail on intestinal permeability and relative abundance of taxonomic units in the gut bacterial community. There was an increase in markers of impaired gut permeability, such as the lactulose/mannitol ratio, plasma endotoxin concentrations, and serum levels of inflammation-related cytokines, following the bacteriophage challenge. We observed significant differences in the alpha diversity of faecal bacterial species and found that richness and diversity index values increased following the bacteriophage challenge. There was a reduction in the abundance of *Blautia, Catenibacterium, Lactobacillus*, and *Faecalibacterium* species and an increase in *Butyrivibrio, Oscillospira* and *Ruminococcus* after bacteriophage administration. These findings provide novel insights into the role of bacteriophages as potentially pathogenic for mammals and their possible implication in the development of diseases associated with increased intestinal permeability.

## Introduction

Our knowledge of the intestinal microbiota has greatly expanded over the last decade, and growing evidence suggests that alterations of the intestinal microbiota are critical pathogenic factors that trigger various polyaetiological diseases associated with increased intestinal permeability and chronic inflammation^1–3^. Previous studies have established a clear correlation between chronic inflammation and a variety of pathologies, including neurodegenerative and cardiovascular diseases, cancer, inflammatory bowel disease (IBD), rheumatoid arthritis, diabetes, aging, among others^4–7^. The primary causes of chronic inflammation in the gut are activation of immunoreactive submucosa and endotoxemia, resulting from the impaired gut barrier function. Understanding the factors that modulate microbiota, leading to an increase in intestinal permeability, is essential to determine the direct and indirect causes of these diseases, which have emerged in Western countries as a major health issue during the past several decades, and to find new approaches to their treatment and prevention.

The microbiota of the human intestinal tract is comprised of bacteria, fungi, and viruses, including bacteriophages. This highly diverse and complex ecosystem is characterised by dynamic stability of each of its components in the context of the host organism. The gut microbiota directly or indirectly modulates all components of the gut barrier function, such as the mucus integrity and transcellular and paracellular transport^8, 9^. The human gut contains approximately 10^15^ bacteriophages, which >10 times of the number of bacterial cells and 100 times of the number of human cells^10^. Although recent studies have shown that the number of free lytic phages in the mammalian gastrointestinal tract is relatively low and prophages are dominant, phages are nevertheless considered important regulators of the microbiota stability^11^. Although it is established that regulators of microbiota can contribute to various pathological conditions in humans, and bacterial viruses are important regulators, there are very few studies of the role of bacteriophages in human health.

Until recently, bacteriophages have been considered not to be harmful to humans since they selectively interact with bacteria and do not affect eukaryotic cells.

Therefore, bacteriophages were used in a number of experimental and clinical therapeutic studies^12, 13^.

Given the critical role of microbiota alterations in the development of increased intestinal permeability, we hypothesised that bacteriophages as important regulators of the microbiota diversity may be implicated in mucosal impairment and thus can indirectly be pathogenic to mammals. We have first revealed that bacteriophages can cause alterations of the microbiota and increase intestinal permeability in a rat model and thus may be harmful for mammals^14^. Furthermore, we have proposed that the primary damage to the microbiota caused by phages should be regarded as a ‘microbiota disease’. We have previously reported that the challenge with bacteriophages is associated with altered intestinal permeability; however, some questions remained unanswered, in particular how microbial composition of the gut microbiota changes and whether it correlates with the increased intestinal permeability and endotoxemia. To investigate these issues, we carried out Illumina sequencing of the V3–V4 region of the 16S ribosomal RNA (rRNA) gene to compare the microbiota composition before and after a bacteriophage challenge and to uncover its role in the increased intestinal permeability and endotoxemia in rats^15^. This information can provide a basis for the crucial next step in the evaluation of bacteriophage implications in poorly treatable human diseases.

## Results

### Bacteriophages increase gut permeability and mediate endotoxemia

The altered microbiota composition and dysfunctional intestinal barrier have emerged as potential triggers of the growing incidence of chronic diseases^1^. To investigate whether bacteriophages can cause a shift in microbiota, leading to increased gut permeability, we used a rat model to measure markers of intestinal permeability and endotoxemia. We examined gut permeability function in animals before and 10 days after the daily challenge with a bacteriophage cocktail using each animal as its own control.

All animals survived the entire duration of the experiment, with no clinical changes in the gastrointestinal tract or stool alteration. We did not detect statistically significant differences in the total weight at baseline and after bacteriophage treatment; however, a trend towards weight loss was registered. After bacteriophage administration, the average weight of each rat was 231 ± 45 compared with 269 ± 28 g in the pre-treatment period. At the same time, all animals showed unkempt hair coats and decreased activity starting from the 5th day of bacteriophage administration, which we believe was due to the induction of endotoxemia and impaired intestinal permeability^16^.

**Figure 1:**
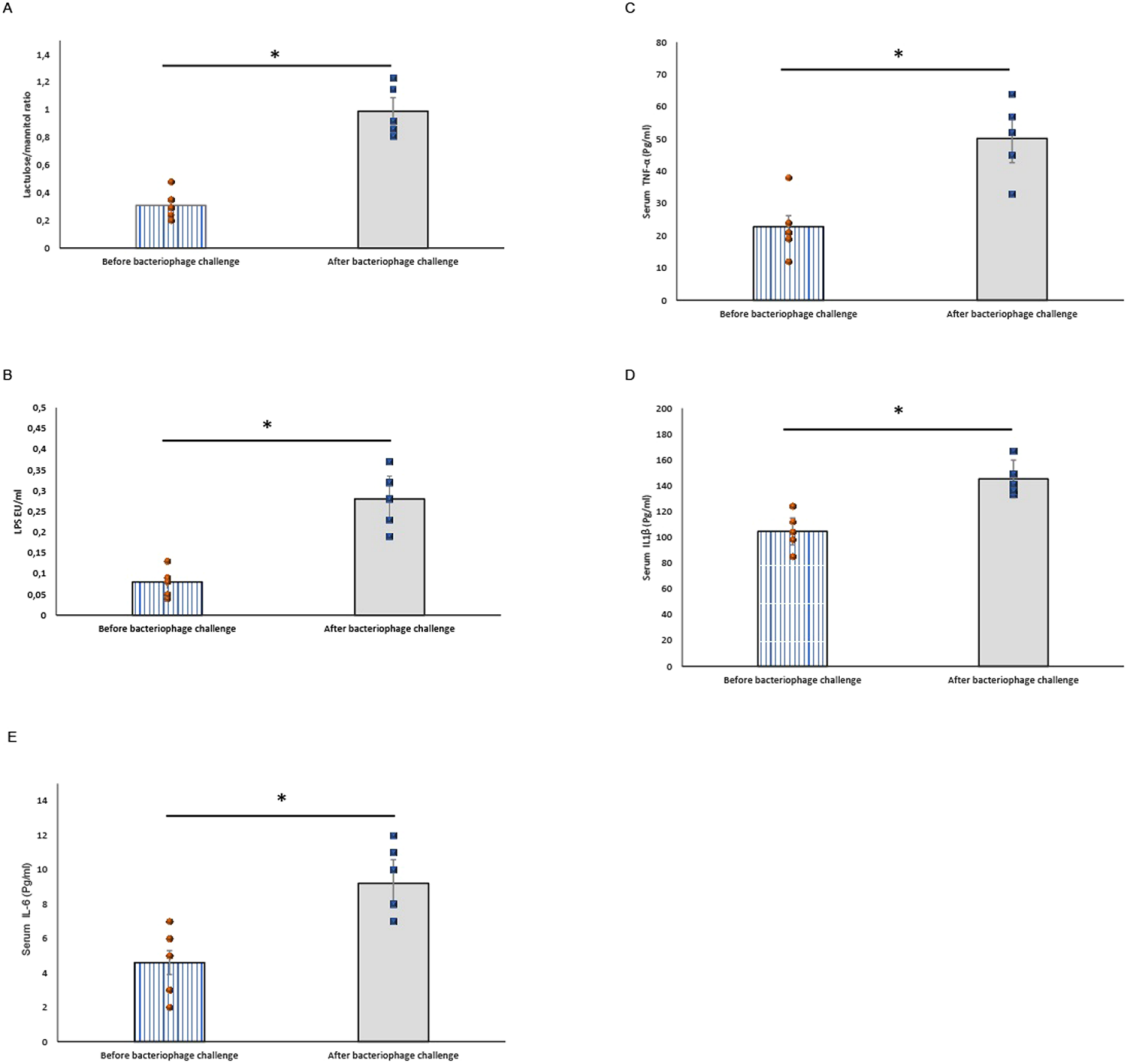
Effects of bacteriophages on intestinal permeability and endotoxemia. Albino Wistar male rats (n = 5) were challenged with a bacteriophage cocktail for 10 days. Each animal was used as its own baseline control. Blood and urine samples were collected from all rats at the same time and subjected to various analytical tests as described in the Materials and Methods section. (**A**) Intestinal permeability expressed as the lactulose/mannitol ratio. Treatment with bacteriophages resulted in a significant alteration of intestinal permeability (p < 0.05). (**B**) Parameters of endotoxemia. Administration of bacteriophages increased the serum LPS levels (p < 0.05). (**C–E**) Serum levels of inflammation-related cytokines: (**C**) TNF-α, (**D**) IL-1β, and (**E**) IL-6. Data are expressed as the means ± standard error of the mean (SEM). The non-parametric paired Wilcoxon signed-rank test was applied to the analysis of pre- and post-challenge differences.

To assess the damage to the gastrointestinal tract, we measured intestinal permeability using a clinical test based on saccharide permeability, which measures the ratio of lactulose to mannitol after oral ingestion of the sugars^17, 18^. The animals showed higher gut permeability following the bacteriophage challenge, with an average post-treatment lactulose/mannitol ratio of 0.99 ± 0.08 compared with that of 0.31 ± 0.05 pre-bacteriophage treatment (p < 0.05) (Fig. 1A). Given that the altered intestinal permeability is commonly associated with endotoxemia, we next determined whether bacteriophages could cause the latter condition^19^. As shown in Fig. 1, the rats exhibited dramatically elevated serum levels of endotoxemia markers after the phage challenge, as compared to the pre-treatment baseline data. We found that the animals had higher levels of plasma lipopolysaccharide (LPS) after the bacteriophage challenge, with an average of 0.28 ± 0.07 endotoxin units (EU)/mL as compared with an average of 0.08 ± 0.02 EU/mL (p < 0.05) before the treatment (Fig. 1B). Accordingly, the levels of serum inflammatory cytokines were also elevated after the bacteriophage challenge^20, 21^. Treatment with phages resulted in a significant increase in the serum concentrations of tumour necrosis factor-alpha (TNF-α), interleukin (IL)-1β, and IL-6 (p < 0.05 compared with the baseline pre-treatment data; Fig. 1C–E).

### Bacteriophages alter intestinal microbial communities

To determine the effects of bacteriophages on the intestinal microbiota, we analysed the gut microbiota composition in the rats before and after the bacteriophage challenge using Illumina-based 16s rRNA sequencing. After quality control filtering, we obtained an average of 147,191 reads of the V3–V4 region of 16S rRNA genes per sample. Operational taxonomic units (OTUs) were defined as a set of sequence reads with the similarity cutoff of 97%^22^.

**Figure 2:**
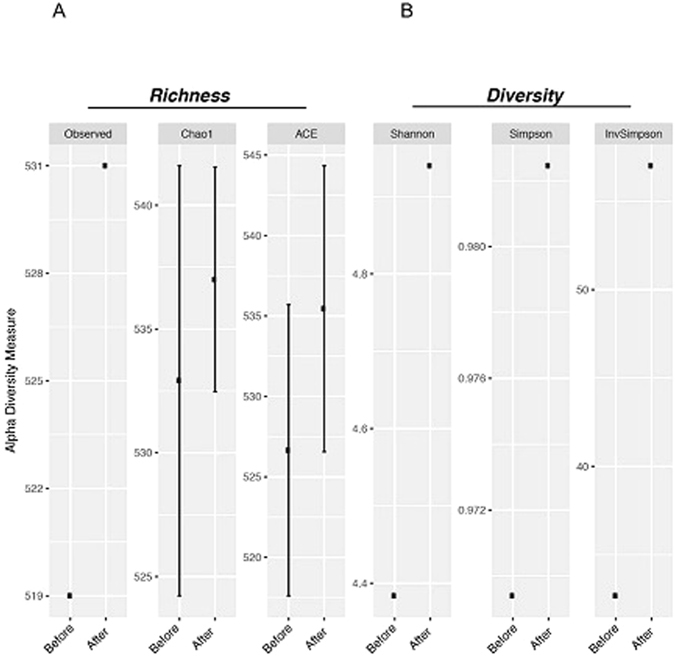
Alpha diversity before and after bacteriophage challenge as calculated by multiple diversity measures. Comparisons of the alpha diversity indexes revealed alterations after the bacteriophage challenge. (**A**) Bacterial richness across the samples was calculated using Chao 1 and ACE. (**B**) Bacterial diversity was evaluated using the Shannon, Simpson, and inverse Simpson parameters. p < 0.05 using *t*-test analysis.

The sequence reads were then used to investigate whether there was an overall gain or loss of diversity by examining the alpha diversity, which represented a difference in the microbiome composition between groups at baseline and after administration of bacteriophages^23^. For this purpose, we calculated the species diversity (Shannon, Simpson, and inverse Simpson indices) and richness estimators (Chao 1 and ACE) pre- and post-phage challenge. We found an increase in the alpha diversity on day 10 of phage administration. The bacterial richness also differed, and the Chao 1 and ACE values, as well as the Shannon, Simpson and inverse Simpson indexes, increased following the phage challenge (Fig. 2). These data demonstrated that the faecal microbiota exhibited a rapid and marked increase in the overall microbial diversity after bacteriophage infection.

**Figure 3:**
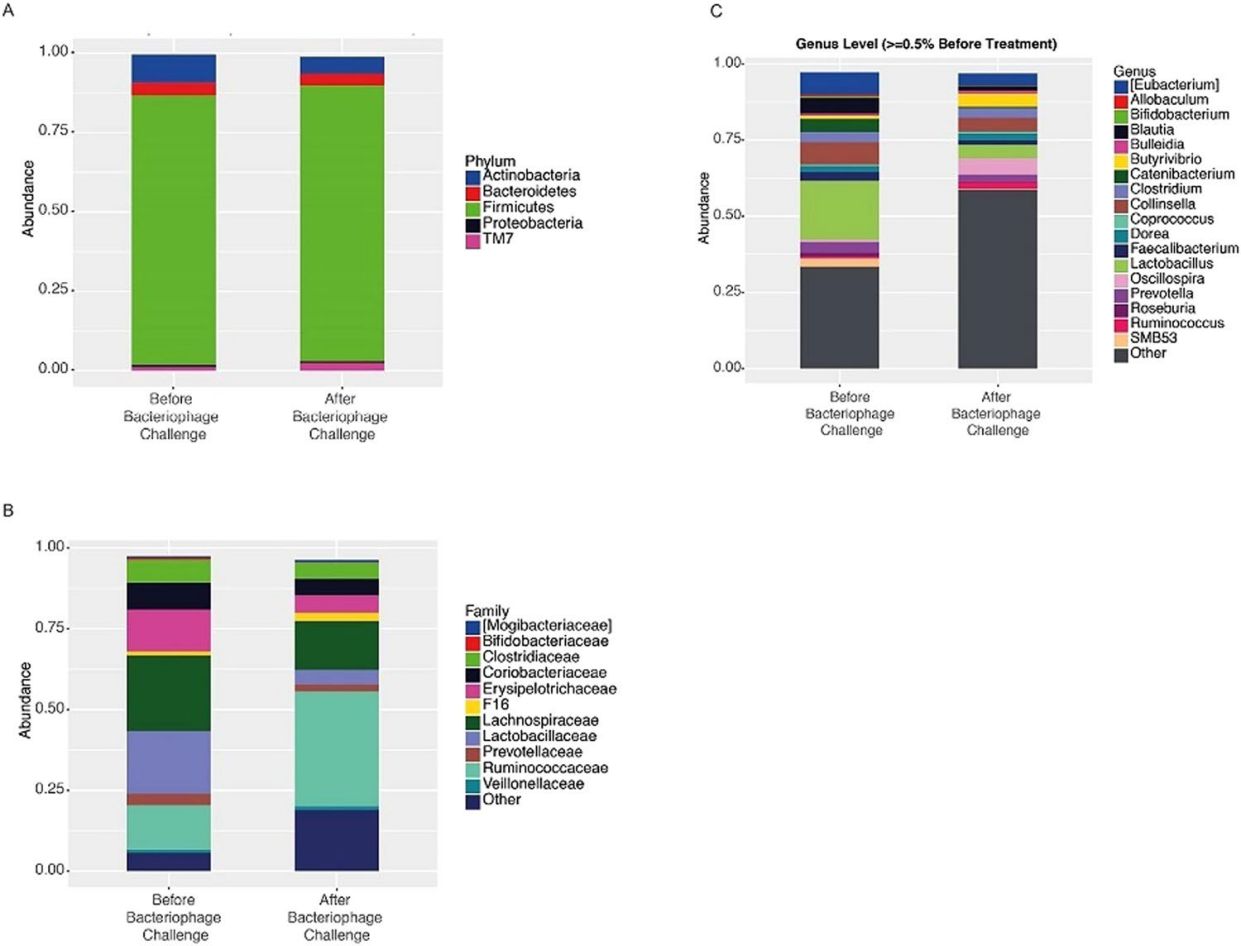
Comparison of relative abundance of predominant genera. Faecal bacterial communities were analysed by high-throughput sequencing of the 16S rRNA gene. Relative abundances of bacterial (**A**) phyla, (**B**) families, and (**C**) genera before and after the bacteriophage challenge at a level of ≥0.5% relative abundance. The term ‘other’ refers to the genera with the minimum abundance of <0.5%.

**Figure 4:**
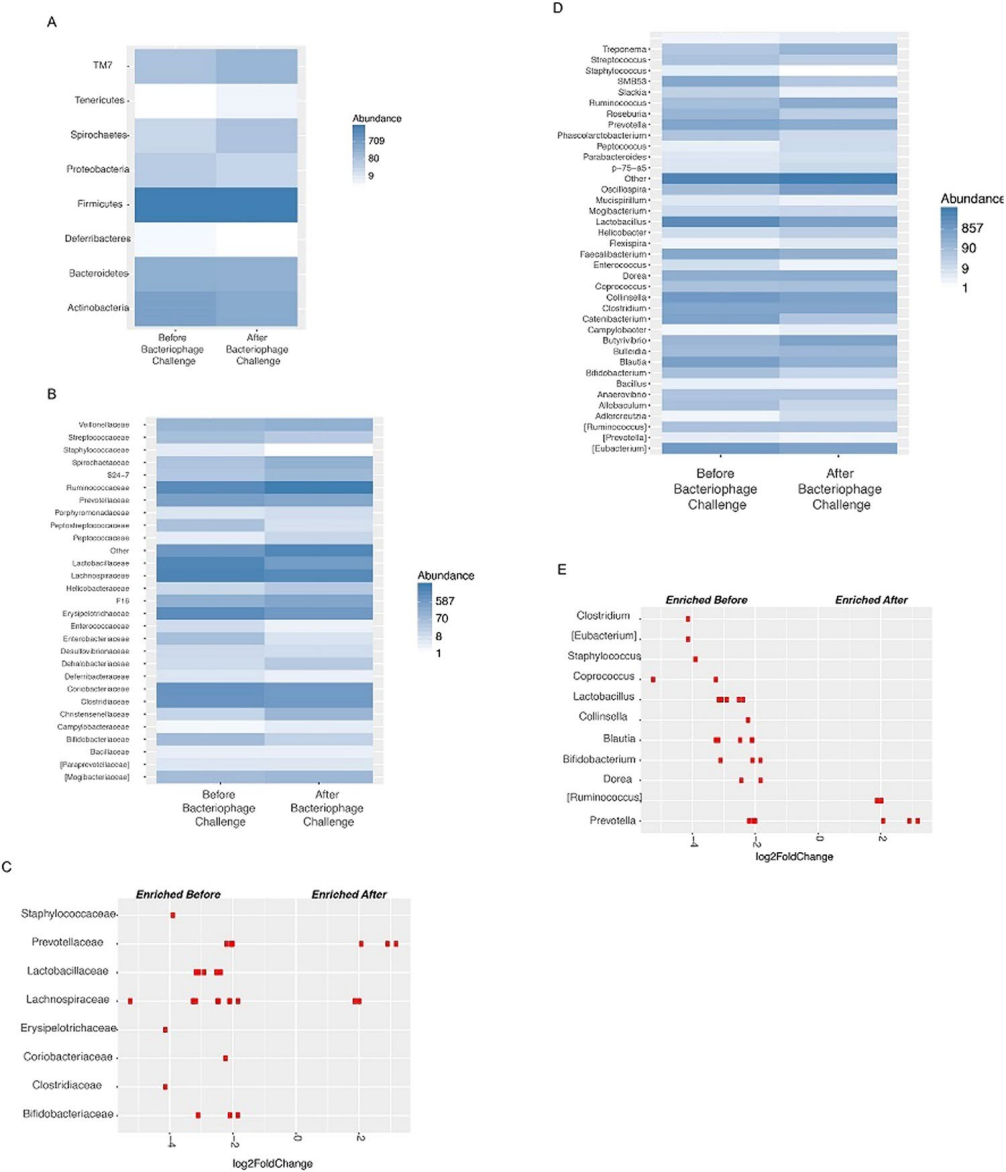
Bacteriophage challenge affects the gut bacterial community. Bacterial OTUs occurring at an abundance of >0.001% before and after bacteriophage challenge. Heatmap of the relative abundances of bacterial (**A**) phyla, (**B**) families, and (**D**) genera. Population scores of enriched taxa of bacterial (**C**) families and (**E**) genera that differed by at least two-fold (i.e. log_2_ = 1) between pre- and post-treatment samples. A positive log_2_-fold change value indicates that OTU is significantly enriched in samples, and a negative log_2_-fold change indicates that OTU is significantly depleted in post-treatment samples.

The taxonomic identity of the reads was analysed using available annotation source databases. With OTUs at a relative abundance of ≥0.5% at the phylum level, *Firmicutes* was the predominant phylum in the untreated and bacteriophage-treated animals (Fig. 3A). At the phylum level, there were no significant changes in the untreated and bacteriophage-treated animals (Fig. 3A). However, analysis of all phylum level (OTUs > 0.001%) abundance data revealed a decreased abundance of *Actinobacteria, Deferribacteres*, and *Proteobacteria* after the bacteriophage challenge. Notably, three phyla, *Spirochaetes, Tenericutes*, and candidate division TM7, increased following bacteriophage treatment (Fig. 4A).

We next examined the compositional changes at the family level with taxa detectable at ≥0.5% (Fig. 3B). Consequently, we detected a decrease in the abundance of *Bifidobacteriaceae*, *Clostridiaceae*, *Erysipelotrichaceae, Lachnospiraceae, Lactobacillaceae*, and *Prevotellaceae*. However, post-bacteriophage samples revealed an increase of *Veillonellaceae*, *Ruminococcaceae*, and unclassified organisms after bacteriophage treatment. More detailed data were generated by the analysis of OTUs detectable at relative abundances of >0.001% (Fig. 4B). We evaluated the families differing by at least two-fold (i.e. log_2_ = 1) between pre- and post-treatment samples (Fig. 4C). The key two-fold alterations following the bacteriophage challenge included a decrease in *Staphylococcaceae, Prevotellaceae, Lactobacillaceae, Lachnospiraceae, Erysipelotrichaceae, Coriobacteriaceae*, *and Bifidobacteriaceae* and an increase in certain members of *Prevotellaceae* and *Lachnospiraceae*.

At the genus level, the treatment with phages resulted in substantial *Blautia*, *Catenibacterium*, *Lactobacillus*, and *Faecalibacterium* depletion and a simultaneous increase in *Butyrivibrio, Oscillospira*, *Ruminococcus* at a relative abundance of >0.5% (Fig. 3B). Notably, the number of unclassified bacteria significantly increased at the genus level.

In addition, we analysed the 16s rRNA data at the species level with OTUs detected at the level of abundance of >0.001% (Fig. 4D). The highest depletion at the genus level, >two-fold (i.e. log_2_ = 1), was registered post-bacteriophage treatment for the genera *Clostridium, Eubacterium, Staphylococcus, Coprococcus, Lactobacillus, Collinsella, Blautia, Bifidobacterium, Dorea*, and *Prevotella*. Simultaneously, certain members of the genera *Ruminococcus* and *Prevotella* were significantly enriched (Fig. 4E).

Overall, we determined that the faecal microbiota of animals exhibited distinct alterations in the bacterial composition following the treatment with bacteriophages.

## Discussion

The aims of the present study were to determine (1) whether bacteriophages could induce alterations of the gut flora, leading to gut leakiness and an impaired intestinal barrier; and (2) whether bacteriophage treatment could increase gut permeability in mammals, sufficient to facilitate endotoxemia and inflammatory responses.

Our knowledge of the particular role of intestinal permeability has greatly increased over the last years^24, 25^. Indeed, with the impaired gut permeability, bacterial antigens cross into the lamina propria, leading to endotoxemia and dysregulation of inflammatory responses, and trigger chronic inflammation, which in turn is an important factor for the development of various polyaetiological diseases^20, 21^. There is increased evidence showing that the concomitant gut barrier dysfunction may play a critical role in diseases such as Crohn disease, IBD, chronic fatigue syndrome, diabetes, autism, cancer, neurodegenerative and cardiovascular diseases^3–7^. At the same time, alterations in the gut microbiota, which is an important regulator of intestinal homeostasis, have been proposed to play a primary role in the impaired gut permeability and deleterious immune activation^2^. Therefore, understanding all the factors that modulate microbiota is essential for the prevention and therapy of a variety of poorly treatable diseases.

Bacteriophages are known to play an important role in microbiota homeostasis and therefore are involved in human health^26^. De Paepe *et al*. described different models including both direct and indirect effects of phages on humans. Indirect models are predominantly realized by the role of phages in dysbiosis, alterations of bacterial properties by transduction, and phage-mediated changes in recognition patterns of bacteria, resulting in an altered immune response. Direct models describe the direct modulation of immunity resulting from phage phagocytosis^27^.

Our previous results have indicated, for the first time, that bacteriophages might be a previously unrecognised cause of diseases in mammals, including humans. To our knowledge, our previous study was the first to indicate that bacteriophage treatment could lead to intestinal hyperpermeability in rodents^14^. We have shown that the challenge of animals with bacteriophages resulted in increased intestinal permeability and elevation of circulating immune complexes. This observation led us to define such alterations as ‘microbiota diseases’, which reflects the nature of the primary impairment caused by bacteriophages.

In the present study, we used the same rat model and treatment regimen to study, in more detail, the changes in the gut microbiota and endotoxemia following bacteriophage treatment. To understand the nature of impaired gut permeability in response to bacteriophage exposure, faecal material was collected from the same animals before and after the phage challenge. We used a bacteriophage cocktail comprised of commercially available and characterised phages active against the *Enterobacteriaceae*, *Staphylococcaceae*, *Streptococcaceae*, and *Pseudomonadaceae* families. Bacteriophages are known to selectively interact with bacteria and not affect eukaryotic cells. Thus, the impaired gut permeability can only be a consequence of altered microbiota^12, 28^.

We found an increased lactulose/mannitol ratio compared with that at baseline, which was determined prior to administration of the bacteriophages, indicating increased intestinal permeability following oral administration of bacteriophages. Our results also showed significantly elevated levels of blood serum endotoxin, TNF-α, IL-1β, and IL-6 compared to those in the pre-treatment period.

Consistent with other studies, which indicated that the alteration of gut microbiota by antibiotics might lead to impaired gut permeability, we showed for the first time that bacteriophages could induce inflammation, most likely through an increase in the circulating endotoxin level, which is likely to be a result of an altered intestinal microbiota and increased intestinal permeability^29^. There are different ways by which a disrupted gut barrier can lead to immune modulation; the most well described is stimulation of inflammatory responses by the intestinal-derived endotoxin (increased serum LPS levels), which subsequently results in elevated levels of inflammatory mediators^18, 30^.

In this study, we found that the bacteriophage challenge affected the microbial alpha diversity. Overall, we detected an increase in the richness and diversity of faecal microbiota, indicating that the total number of bacterial species increased after phage treatment compared to the baseline pre-treatment data. The increased richness of intestinal microbiota is considered one of the signatures of a leaky gut and a feature of intestinal inflammation. Similar patterns have been described in other diseases associated with increased intestinal permeability^31, 32^. Our study identified bacteria whose abundance changed following the bacteriophage challenge, and this alteration resulted in increased intestinal permeability, since phages selectively target bacteria with no effect on mammalian cells^33, 34^.

At the genus level, samples collected after the treatment with bacteriophages showed increases in *Oscillospira* and *Butyrivibrio* and decreases in *Lactobacillus* and *Faecalibacterium*; however, only *Lactobacillus* showed a two-fold change. The decreases in *Lactobacillus and Faecalibacterium* represent an important pattern, regarded as a signature of impaired gut permeability and inflammation^35, 36, 37^. Both bacterial genera are known to be beneficial for mammals, and studies have shown that their depletion is associated with barrier abnormalities. In a number of studies, both bacterial genera, which are considered effective anti-inflammatory microorganisms, were shown to restore the function of the intestinal barrier. Thus, *Lactobacillus rhamnosus* CNCM I-3690 and the commensal bacterium *Faecalibacterium prausnitzii* A2-165 exhibited similar protective effects against induced barrier hyperpermeability in mice.

It is interesting to note that the increase in relative abundance of the butyrate-producing bacteria *Butyrivibrio*, known to lead to decreased expression of proinflammatory cytokines and suppression of proinflammatory responses^38, 39^. *Butyrivibrio* are known to reduce bacterial translocation by potentiation of mucin synthesis, are beneficial for tight junctions, and are suggested to suppress intestinal hyperpermeability^40^.

Of note, the bacteriophages used in this study had no direct impact on the genera *Lactobacillus*, *Faecalibacterium*, and some others whose abundance was significantly altered. This is consistent with previous studies, which have shown that microbiota is characterized by temporal stability and dynamic equilibrium and its alterations result in complex and poorly predicted responses and consequences^41^.

It is likely that the decrease in the abundance of certain genera, which were directly affected by the phages, triggered a cascade of microbiota alterations, eventually leading to the depletion of *Lactobacillus* and *Faecalibacterium* species. This may be the result of alteration of the abundance of species having symbiotic associations with *Lactobacillus* and *Faecalibacterium* or of outgrowth of species having inhibitory interactions with these two species^42^.

An example of possible alterations of symbiotic properties is the decreased relative abundance of *Streptococcus* spp that are known to have a mutualistic relationship with certain *Lactobacillus* spp as a result of the direct impact of phages used in this study^43^.

In contrast, one of the possible inhibitory pathways may involve increased abundance of *Helicobacter* spp., whose antagonistic effects towards Lactobacillus were previously described^44, 45^. In turn, the abundance of Helicobacter is likely to be increased by the decrease of *Enterobacteriacea*, which is directly affected by the bacteriophage cocktail^46^.

On the other hand, participation of these species in the gut balance is well known, while relative contributions of other bacteria, whose abundance also changed in this study and which could regulate the intestinal barrier function, may be underestimated. Therefore, at this point the exact pathway of microbiota alteration that leads to gut leakiness remains unknown. However, this was not the goal of our study since bacteriophages are known to specifically target specific bacterial species, and therefore, other types of phages may lead to different microbiota alterations and consequences for the macroorganism.

The experimental model used allows to minimize possible direct effect of used phages on mammalian intestinal epithelium as there are numerous of available receptors of specific bacteriophage receptors on the surface of the bacteria of rats’ microbiota.

Collectively, our results warrant further research on microbiota diseases and previously underestimated consequences related to bacteriophage exposure in mammals, including humans. We suggest that the global distribution of bacteriophages, their high prevalence in the outer environment and microbiota, and their potential to alter gut permeability, which was revealed in this study, should drive future research regarding phage implications in a variety of emerging diseases. Although it is not possible to determine, based on this study, if the observed alterations occur in the natural environment, one may assume that under certain conditions, bacteriophage infections could be contagious and spread among mammalian hosts, triggering polyaetiological conditions. Follow-up studies on the role of bacteriophages should be considered for better understanding of the implication of phages in mammalian diseases and the spread of poorly treated diseases, associated with alterations of microbiota, in humans.

## Materials and Methods

### Animals

Healthy adult male Wistar rats (n = 5; 12-week-old, 240–280 g) were maintained in individual cages in a P3 room under a 12-h light/dark cycle, at a temperature of 22 to 25 °C and 60 ±  5% atmospheric humidity. All animals had free access to food and water according to the Guide for the Care and Use of Laboratory Animals^47^. An ethical approval was granted by the Human Microbiology Institute Ethics Committee (B12/2016).

The animals were monitored daily for any changes of their activity, behaviour, and general health status, including the weight loss, ruffled coat, wheezing or abnormal respiration, and the presence of unformed faeces. Body weights of all animals were measured using a digital balance (Mettler-Toledo, Inc., Columbus, OH, USA).

### Reagents

Lactulose (L7877, Sigma–Aldrich) and mannitol (M8429, Sigma–Aldrich) were utilised for all arms of the study.

### Phages and experimental model

A commercial bacteriophage cocktail against *Enterobacteriaceae*, *Staphylococcaceae*, and *Streptococcaceae* families was used to induce microbiota diseases. It included a *Salmonella* bacteriophage cocktail from Microgen (Moscow, Russia; product batch number H20), containing bacteriophages against *S*. Paratyphi*, S*. Typhimurium*, S*. Heidelberg*, S*. Newport*, S*. Choleraesuis*, S*. Oranienburg, *S*. Infans*, S*. Dublin*, S*. Enteritidis*, S*. Anatum, and *S*. Newlands. Pyobacteriophage, another polyvalent commercial phage cocktail from Microgen (product batch number 4), contained phages against seven bacterial species, *Staphylococcus aureus*, *Streptococcus pyogenes*, *Proteus mirabilis*, *Proteus vulgaris*, *Pseudomonas aeruginosa*, *Klebsiella pneumoniae*, and *Escherichia coli* ^48^. Phages (1.5 mL, 1 × 10^6^ plaque-forming units/mL of each phage cocktail) were added to drinking water according to the manufacturer’s instruction and administered orally for 10 days. Each animal was used as its own control before the bacteriophage challenge.

### Measurement of gut permeability *in vivo*

Intestinal permeability in rats was assessed after 8-h fasting as previously described^49^. In brief, animals received 2.0 mL of a solution containing 120 mg of lactulose and 80 mg of mannitol by oral gavage. Two tests of intestinal permeability were performed in each animal, at baseline and 10 days after daily phage administration. Rats were placed individually in metabolic cages, and urine was collected for 24 h. Then, we measured the lactulose/mannitol ratio using gas chromatography as described previously^50^.

### Serum markers of inflammation

Levels of TNF-α, IL-6, and IL-1β were measured in the serum using respective commercial rat-specific enzyme-linked immunosorbent assay (ELISA) kits (BioLegend, San Diego, CA, USA) according to the manufacturer’s instructions. LPS was measured using a Pyrochrome Limulus Amebocyte lysate kit (Associates of Cape Cod, Inc., East Falmouth, MA, USA) according to the manufacturer’s instructions.

Great care was taken to ensure aseptic collection and to avoid contamination with environmental LPS.

Venous blood samples were collected atraumatically and aseptically, with the skin shaved and sterilized with alcohol swabs. In brief, blood specimens were collected under pyrogen-free conditions and stored in LPS-free vials (Eppendorf, Germany) on ice^51^.

Plasma was centrifuged, transferred to a glass tube, and stored at −20 °C until analysis. Optical densities were measured by ELISA and read at 405 nm.

### Comparative faecal microbiome analysis

Faecal samples were collected at baseline and after 10 days of the bacteriophage challenge into a sterile container and immediately stored at −80 °C until further processing. Bacterial DNA extraction was performed using a QIAamp stool DNA mini kit according to the manufacturer’s instructions (Qiagen, Germany). Sequencing libraries of the V3–V4 region were prepared according to the Illumina MiSeq system instructions^52^. In brief, the V3 and V4 regions of the 16S bacterial rRNA gene were amplified using a two-step polymerase chain reaction (PCR) protocol with V3 and V4 region primers (forward: 5′-TCGTCGGCAGCGTCAGATGTGTATAAGAGACAGCCTACGGGNGGCWGCAG-3′; reverse: 5′-GTCTCGTGGGCTCGGAGATGTGTATAAGAGACAGGACTACHVGGGTATCTAATCC-3′) for the first PCR and Nextera XT index primers for the second PCR. Amplicons were cleaned using AMPure XP magnetic beads, and then, Illumina sequencing adapters and dual-index barcodes were added to each amplicon. Libraries were assessed with the Qubit dsDNA HS assay kit (Thermo Fisher Scientific) and TapeStation high sensitivity D1000 ScreenTape (Agilent) and normalised and sequenced on an Illumina MiSeq instrument using a MiSeq reagent kit v2 (500 cycles). Data was analysed with the MiSeq Reporter software Metagenomics workflow v2.5.1.3 (Illumina). The average number of reads was approximately 147,191 per sample.

### Analysis of sequencing data

FASTQ files generated by Illumina sequencing were qualitatively evaluated using FASTQC. Adaptor contamination and low-quality reads were detected with Trimmomatic^53^. Raw data were quality-filtered to remove sequences with <200 nucleotides or containing ambiguous bases, uncorrectable barcodes, and homopolymer runs of more than six bases. Sequences were grouped into OTUs with a 97% threshold of pairwise identity^54^. For clarity and visualisation purposes, the most abundant sequences with a relative abundance of ≥0.5% within each taxa were designated as ‘representative sequences’. OTUs that had a mean relative abundance of >0.001% were designated as ‘total sequences’ and were analysed separately. Representative and total sequences were assigned at different taxonomic levels (from phylum to genus) to bacterial SILVA datasets^54–57^.

The QIIME pipeline was used for quality filtering of DNA sequences, chimera removal (using the USEARCH software), taxonomic assignment, and calculation of the alpha diversity as previously described^58, 59^. Downstream data analysis and calculation of diversity metrics was completed in R3.3.2 using ggplot2 and phyloseq libraries and DESeq. 2 for calculating the log fold change^60^. The abundance-based coverage estimator (ACE), Chao 1 richness estimator, Shannon, Simpson, and inverse Simpson diversity indices were calculated using the phyloseq R library^61^.

### Statistical analysis

All statistical analyses were performed using the statistics package Statistica for Windows (version 5.0). Results are reported as the mean ± SEM for each group. The non-parametric paired Wilcoxon signed-rank test was applied to the analysis of pre- and post-challenge differences. A value of p < 0.05 was considered significant.
